# Functional MRI neurofeedback training on connectivity between two regions induces long-lasting changes in intrinsic functional network

**DOI:** 10.3389/fnhum.2015.00160

**Published:** 2015-03-30

**Authors:** Fukuda Megumi, Ayumu Yamashita, Mitsuo Kawato, Hiroshi Imamizu

**Affiliations:** ^1^Advanced Telecommunications Research Institutes InternationalKyoto, Japan; ^2^Graduate School of Information Science, Nara Institute of Science and TechnologyIkoma, Japan; ^3^Institute of Cognitive Neuroscience, University College LondonLondon, UK; ^4^Department of Systems Science, Graduate School of Informatics, Kyoto UniversitySakyo-ku, Japan; ^5^Center for Information and Neural Networks, National Institute of Information and Communications Technology and Osaka UniversitySuita, Japan

**Keywords:** functional MRI neurofeedback, intrinsic functional network, resting state functional connectivity, long-lasting changes, default mode network

## Abstract

Motor or perceptual learning is known to influence functional connectivity between brain regions and induce short-term changes in the intrinsic functional networks revealed as correlations in slow blood-oxygen-level dependent (BOLD) signal fluctuations. However, no cause-and-effect relationship has been elucidated between a specific change in connectivity and a long-term change in global networks. Here, we examine the hypothesis that functional connectivity (i.e., temporal correlation between two regions) is increased and preserved for a long time when two regions are simultaneously activated or deactivated. Using the connectivity-neurofeedback training paradigm, subjects successfully learned to increase the correlation of activity between the lateral parietal and primary motor areas, regions that belong to different intrinsic networks and negatively correlated before training under the resting conditions. Furthermore, whole-brain hypothesis-free analysis as well as functional network analyses demonstrated that the correlation in the resting state between these areas as well as the correlation between the intrinsic networks that include the areas increased for at least 2 months. These findings indicate that the connectivity-neurofeedback training can cause long-term changes in intrinsic connectivity and that intrinsic networks can be shaped by experience-driven modulation of regional correlation.

## Introduction

Spatial and temporal correlations in spontaneous brain activity are generated by the underlying connectivity of brain networks (Ringach, 2009). An increasing number of functional neuroimaging studies have used resting-state functional connectivity magnetic resonance imaging (rs-fcMRI), which quantifies correlations in low-frequency fluctuations of spontaneous blood oxygen level-dependent (BOLD) signals during rest (Biswal et al., 1995; Raichle et al., 2001; Fox and Raichle, 2007). Independent component analysis (ICA) of data in thousands of task-driven activation studies has demonstrated co-activation networks that were strikingly similar to networks estimated by spatial ICA of the resting state fMRI (Smith et al., 2009; Laird et al., 2011). These studies indicate that the repertoire of functional networks is continuously and dynamically activated even during “rest” and that the dynamics represent the brain architecture. It has been suggested that intrinsic connectivity investigated by rs-fcMRI reflects closely, though not exactly, the structural or anatomical connectivity (Vincent et al., 2007; Greicius et al., 2008; Honey et al., 2009) and that abnormality in rs-fcMRI is related to many psychiatric and neurological disorders (Broyd et al., 2009).

An interesting aspect of functional connectivity is that experiences in daily life (Fair et al., 2007) and behavioral training, including motor learning (Albert et al., 2009; Taubert et al., 2011; Vahdat et al., 2011) and perceptual learning (Lewis et al., 2009), lead to changes in intrinsic connectivity (Fair et al., 2007; Lewis et al., 2009) and functional networks. However, little is known about a possible cause-and-effect relationship between a specific change in regional-activation correlations and a long-term change in intrinsic networks. Here, we propose and directly test the hypothesis that spontaneous brain activity is shaped in an experience-driven manner, where the temporal correlation at rest between two brain regions is increased when they are simultaneously activated or deactivated. In the current study, “functional connectivity” is defined as a temporal correlation between activations in two brain regions, which is calculated from BOLD time courses. We assume that an absolute value of correlation reflects the strength of functional connectivity, e.g., a large negative (*r* ≈ −1.0) or positive (*r* ≈ +1.0) correlation means a strong connectivity while a correlation near zero (*r* ≈ 0.0) means a weak connectivity.

To examine our hypothesis, we developed a straightforward method for inducing regionally specific correlated activations based on online fMRI neurofeedback (see reviews: Weiskopf et al., 2003; deCharms, 2007; Sulzer et al., 2013). Previous fMRI neurofeedback methods successfully up- or down- regulated single region-of-interest (ROI) activation (deCharms et al., 2005; Subramanian et al., 2011); furthermore, these studies were extended to control voxel patterns of spatial activity (Shibata et al., 2011) and multiple brain regions (Robineau et al., 2014). These studies have proven that online neurofeedback training can be an effective method to manipulate brain activation at various levels. In our connectivity-neurofeedback training, the temporal correlation of activation in two specific regions (see below) during a motor imagery task for 14 s was calculated. Subjects were informed of the correlation immediately after each trial as an intermittent feedback. A monetary reward was given in proportion to the increment of the correlation. Through repetition of a few hundreds of trials over 4 days, subjects learned to increase the correlation between the regions in a trial-and-error manner while guided by the reward without conscious understanding of the meaning of feedback signals (Weiskopf et al., 2004; Bray et al., 2007; Shibata et al., 2011).

Recently, the effect of single-ROI regulation was investigated on functional connectivity and networks including rs-fcMRI (Ruiz et al., 2011; Harmelech et al., 2013; Scheinost et al., 2013; Scharnowski et al., 2014). Furthermore, the importance of connectivity-neurofeedback for improvement of cognitive functions and psychiatric disorders was suggested in a review article (Ruiz et al., 2014), and manipulation of local network dynamics was examined through an online evaluation of hypothesized connectivity models (Koush et al., 2013). These studies suggest that neurofeedback training induces changes in functional connectivity. We were specifically interested in the changes in correlation across intrinsic networks, and thus we selected two regions from distinct intrinsic networks.

Specifically, we selected the left primary motor cortex (lM1) from motor/visuospatial network group (MVN) and the left lateral parietal cortex (lLP) from default mode network (DMN) (Callard and Margulies, 2014) according to a meta-analysis study (Laird et al., 2011). A number of rs-fcMRI studies have consistently shown that the DMN and some of networks related to MVN (such as the dorsal attention network) are negatively correlated (Fox et al., 2005; Fransson, 2005; Biswal et al., 2010). The genotype of the dopamine transporter affects the degree of the negative correlation (Gordon et al., 2012). The existence of consistent DMN across mammalian species indicates an evolutionary organization of the negative correlation (Rilling et al., 2007; Vincent et al., 2007; Lu et al., 2012). If our method can change this correlation, it suggests that trainings and experiences modify connectivity largely determined by genetic and evolutional factors, and that the connectivity-neurofeedback training will contribute to educations and medical treatments.

## Materials and methods

### Participants

Thirty-three healthy subjects (23 males and 10 females, aged 19–43 years) participated in this experiment. All subjects were right-handed according to the Edinburgh inventory (Oldfield, 1971). Following a previous neurofeedback study that uses a neurofeedback-training group and several control groups (deCharms et al., 2005), we randomly assigned subjects to a test group (*n* = 12) or one of two control groups (a sham-feedback group: *n* = 12 and a tapping-imagery group: *n* = 9; see below), each of which had more than eight subjects. There was no significant difference in age [*F*_(2, 30)_ = 0.34, *p* = 0.72, n.s] and gender balance [*F*_(2, 30)_ = 2.03, *p* = 0.15, n.s.] across groups. The Institutional Review Board of Advanced Telecommunications Research Institute International (ATR) approved this study. All subjects gave written informed consent.

### MR image acquisition

Images were obtained using a Siemens MAGNETOM Trio (3 Tesla) scanner. BOLD signals were measured using echo planar imaging (EPI) sequence (volume repetition time, 2 s; echo time, 30 ms; flip angle, 80°). The entire brain was covered in 33 axial slices (3.5-mm thickness; no gap), voxel size was 3 × 3 × 3.5 mm, and field of view was 192 × 192 mm. T1-weighted structural images were acquired with 1 × 1 × 1-mm resolution. T2-weighted structural images were acquired on each day with 1 × 1 × 3.5-mm resolution.

### Neurofeedback training protocol

**Figure 1:**
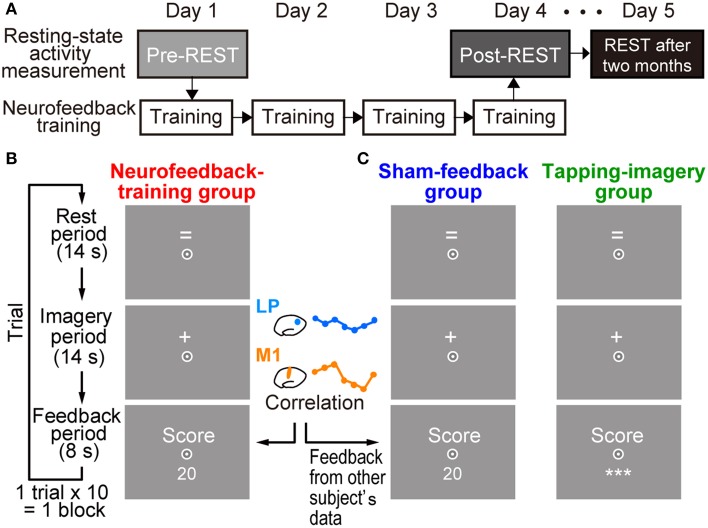
**Experimental procedures. (A)** Measurement of resting state activity and neurofeedback training. Resting state activity measurement (pre-REST) was followed by neurofeedback training for 4 days. Resting state activity was also measured at the end of the fourth day of training (post-REST) and 2 months after the training (REST after 2 months). **(B)** Timeline and displays for subjects in a trial during training in the neurofeedback-training group. After a rest period while the “=” sign was presented on the screen, subjects were instructed to produce tapping imagery during the imagery period while the “+” sign was presented. They received a numerical feedback score (e.g., 20) that represented an increased correlation of activation in the left motor cortex (lM1) and the left lateral parietal region (lLP) during the imagery period (see Materials and Methods). **(C)** Displays for control groups. Subjects were also required to produce tapping imagery. The sham-feedback group received feedback, which was calculated from the activity of another subject. For the tapping-imagery group, the score value was not presented to the subjects; instead, three asterisks (***) were presented on the screen. Subjects were required to fix their eyes to a circle at the center of the screen.

Subjects in the test group received neurofeedback training to increase temporal correlation of BOLD signals between two target ROIs: lM1 and lLP (see Figure 4A). Each subject received training for 4 days (white boxes in Figure 1A). Subjects underwent 5.0 blocks of training per day on average (SD: 1.32), with each block consisting of 10 trials, for a total of 20.0 (SD: 1.73) blocks (see below for the other groups). A trial in each block began with a rest period of 14 s, during which the “=” sign was presented on the screen (Figure 1B). When the sign changed to “+,” subjects performed the tapping imagery task for 14 s (imagery period). Subjects were instructed to imagine tapping their thumbs with their fingers randomly as fast as possible during the imagery period. They were instructed to produce kinetic imagery related to tapping, rather than attempt visual imagery of tapping fingers, and not to overtly move their hands during the task. After the imagery period, a feedback score calculated by the online MRI system (see below for calculation of feedback score) was presented on the screen (feedback period). As noted below, the score was determined from the correlation between two regions.

### Online calculation of feedback score

Temporal correlations between BOLD signals were averaged within the target ROIs during the imagery period, and a numerical score was presented on the screen in the feedback period (right panel of Figure 1B). The score indicated an increment of the correlation from that in the initial block on the first day (see below). We used MATLAB (The MathWorks, Inc.) for online processing of fMRI data. We also used realignment modules of SPM8 (Wellcome Department of Cognitive Neurology, London, UK; http://www.fil.ion.ucl.ac.uk/spm). The software ran on a computer that accessed data files in the MRI system. Each volume of the functional image (EPI) was realigned in real time to the first volume obtained on the first training day. Seven volumes were obtained during an imagery period in each trial, but the first volume was discarded and one volume from the feedback period was added. One may argue that a one-volume shift (2 s) is not enough to fully compensate the hemodynamic delay (4–8 s). However, in a previous fMRI neurofeedback study with an intermittent feedback paradigm (Bray et al., 2007), the first 2 s of each imagery period was eliminated from the feedback signal computation, and participants were able to learn to regulate their own brain activity. We followed their approach and this enabled us to minimize the delay of feedback to participants. BOLD signal time courses were extracted from the lM1 and lLP ROIs in these volumes. To remove low-frequency trends in the BOLD signal, a high-pass temporal filter (cutoff frequency of 0.06 Hz) was applied to the time courses.

Using the filtered time courses, the feedback score in the imagery period of the *i*-th trial was calculated as:

(1) $$Score_{i}=\frac{\left(Correlation_{i}-Correlation_{Initial}\right)}{1+\left|Correlation_{Initial}\right|}\times 100$$

Here, *Correlation_i_* represents the correlation of time courses averaged within the ROIs. *Correlation_Initial_* is the correlation averaged over trials in the initial block of the first training day. We added “1” in the denominator to keep the score range between −100 and +100. Thus, the score represents increments of the correlation value in each trial compared to the value in the first training block. The purpose of using initial score as a baseline was to compensate individual differences in the initial correlation and to keep participants motivated. For instance, if the correlation changes from *r* = −0.6 to −0.2, the above score increases from 0 to 25. We note that the score was designed so that positive reward is given in all of the following three cases: (i) a positive correlation increases in its magnitude (e.g., from 0.2 to 0.6); (ii) a negative correlation changes to a positive correlation (from −0.2 to 0.2); and (iii) a negative correlation increases but stays negative, thus decreasing in absolute value (e.g., from −0.6 to −0.2). We note that the objective of our connectivity neurofeedback training is to increase the correlation and not to increase the absolute value of the correlation, which has sometimes been termed “connectivity” elsewhere. The score was calculated immediately after the acquisition of the first volume in a feedback period (2 s). Preprocessing and score calculation were completed within 2 s. Thus, subjects received the score within 4 s after the end of the imagery periods.

Subjects were instructed to imagine tapping their thumbs with their fingers randomly as fast as possible during the imagery period, with the aim of increasing the score that reflects their imagery performance. During the initial block of the first day, no score was provided to the participants. They were informed that we compute the baseline of their brain-training performance in the first training session. The post-experiment debriefing (Supplementary Text S1) indicated that all subjects performed the mental motor imagery task during training. A standard Statistical Parametric Mapping (SPM) analysis revealed activities in motor-related areas, especially in the left supplementary motor area (SMA) and the premotor area (PM; see Supplementary Figure S1), thus supporting the subjects' reports. We confirmed that subjects had no knowledge of ROIs or how the score was calculated.

Subjects received additional monetary reward in proportion to their total score on each day. Subjects were instructed to increase their score by producing tapping imagery, and they were informed that a monetary reward (up to 2000 JPY) would be paid in proportion to their score. At the end of each block, the averaged score within the block was presented on the screen. We showed a negative score when the temporal correlation between the ROIs dropped in relation to the baseline. We did not decrease monetary reimbursement when negative scores were shown to participants.

### Control groups

Subjects in the two control groups were trained using a similar procedure but without the proper neurofeedback information (Figure 1C). A sham-feedback group received scores and reward, but the scores' time series was taken from another subject randomly chosen from the neurofeedback-training group. We used the score time-series of all subjects in the neurofeedback-training group (*n* = 12) for sham-scores in the sham-feedback group (*n* = 12). Each subject in the sham-feedback group saw a time-series from one randomly selected different subject in the neurofeedback-training group. No other subject in the sham-group was given the same feedback time-series of that subject in the neurofeedback-training group. As explained later, the scores in the neurofeedback-training group increased as training proceeded. Thus, the sham-feedback control group was used to examining whether a spurious increase in score/reward, which does not correctly reflect the actual correlation between the two ROIs in one's own brain, could induce increased correlation between the ROIs. If the sham-feedback group does not show changes in functional connectivity, we can exclude any mechanism that explains the changes in functional connectivity solely by a combination of mental motor imagery and slowly increasing score/reward. A tapping-imagery group (*n* = 9) was instructed to perform the imagery task without the feedback score. In this group, we examined whether the repetition of mere tapping imagery could increase the correlation between the ROIs. Subjects in the sham-feedback and tapping-imagery groups underwent a total of 19.5 (SD 1.85) and 20.56 (SD: 1.07) blocks, respectively.

### ROI definition for neurofeedback training

We determined two ROIs for calculation of the feedback score. We selected the lM1 as one of the two ROIs. This is because consistent temporal changes in BOLD time courses with significant amplitudes are necessary for calculation of a reliable temporal correlation of the two regions during the 14-s training periods. We followed previous neurofeedback studies (deCharms et al., 2004; Bray et al., 2007) that successfully induced large BOLD modulation in the lM1 by asking subjects to imagine finger movements. The lM1-ROI was defined as Brodmann area 4 according to the anatomical map in PickAtlas (http://fmri.wfubmc.edu/software/PickAtlas) (Lancaster et al., 1997; Maldjian et al., 2003).

As the other ROI, we selected a region in the DMN because activity in DMN regions is expected to negatively correlate with that in the lM1 during the motor imagery task. We found a negative correlation before the neurofeedback training during rest as shown in the Results section. We aimed to induce a marked change in the correlation. We adopted a region in the left lateral parietal cortex (lLP) as the nearest DMN region to lM1. This is because functional connectivity between closer regions is on average higher than that between distant regions (Bullmore and Sporns, 2012), and a strong negative correlation is required to compute a reliable feedback score within a short time interval (14 s) at least for the initial stage. Another reason for selection of close regions is to minimize the temporal gaps between activation in the two regions. MRI cannot acquire BOLD signals simultaneously over the entire brain but instead acquires signals sequentially slice by slice. Thus, the longer distance between the two regions may increase the greater temporal gap. Anatomically, lLP is a part of Brodmann's area 39 and the posterior-lateral part of the intraparietal-sulcus region (see Table 1 for coordinates). Previous studies reported lLP as a part of the DMN; in particular, rs-fcMRI studies indicated anti-correlation with regions such as the dorsal attention networks (Fox et al., 2005; Van Dijk et al., 2010), and a meta-analysis of attention-demanding experiments reported a consistent decrease in activity during task performance (Shulman et al., 1997). In accordance with previous studies of the DMN (Fox et al., 2005; Fox and Raichle, 2007), we refer to this region as the “lateral parietal (LP).” The lLP-ROI was defined as a sphere with a 7.5-mm radius centered at (*x, y, z*) = (−45, −67, 36) in the Montreal Neurological Institute standard brain coordinates (MNI; Montreal, Quebec, Canada) according to a previous study on brain networks (Biswal et al., 2010).

**Table 1:** **Sixteen regions of interest for analysis of resting state fMRI**.

**Network**	**Label**	**Anatomical region**	**Brodmann area/MNI coordinates**	**Averaged volume in individual space (mm^3^)**
Motor/visuospatial network	lM1	Left primary motor cortex	Area 4	3307
	rM1	Right primary motor cortex	Area 4	3906
	lSMA	Left supplementary motor area	Area 6	13,302
	rSMA	Right supplementary motor area	Area 6	14,662
	lIPS	Left intra-parietal sulcus	(−25, −57, 46)	1475
	rIPS	Right intra-parietal sulcus	(25, −57, 46)	1489
	lFEF	Left frontal eye field	(−25, −13, 50)	1566
	rFEF	Right frontal eye field	(25, −13, 50)	1607
Default-mode network	lLP	Left lateral parietal region	(−45, −67, 36)	1533
	rLP	Right lateral parietal region	(45, −67, 36)	1608
	PCC	Posterior cingulate cortex	(−5, −49, 40)	1533
	MPF	Medial prefrontal cortex	(−1, 47, −4)	1418
Control (visual and auditory networks)	lV1	Left primary visual cortex	Area 17	1575
	rV1	Right primary visual cortex	Area 17	1701
	lA1	Left auditory cortex	Areas 41 and 42	2014
	rA1	Right auditory cortex	Areas 41 and 42	2043

Because these ROIs were defined in the standard brain, we identified corresponding voxels in the functional images of individual subjects' brains using a deformation module in SPM8. We took several volumes of functional images for this purpose at the beginning of the experiment on the first training day, and we used the identified voxels as ROIs for calculating scores in the subsequent training blocks.

### Measurement of rs-fcMRI

For all subjects, we measured rs-fcMRI before (pre-REST), immediately after (post-REST), and more than 2 months (REST after 2 months) after the training (gray/black boxes in Figure 1A) to investigate the effects of training on intrinsic functional connectivity at rest. Rs-fcMRI was recorded for 5 min (152 volumes) in a run with the standard protocol (Van Dijk et al., 2010). Subjects were instructed to gaze at a fixation point on a screen, not to move during the measurements, and not to recall or rehearse what they did during neurofeedback training. The major frequency of rs-fcMRI was below 0.05 Hz while a 0.06-Hz high-pass filter was applied to signals in neurofeedback training. Therefore, the rs-fcMRI provided an index of training effect that was independent of the feedback score (see Supplementary Figure S2 for spectrum density analysis). This indicates that the recall or rehearsal unlikely affects rs-fcMRI (see Supplementary Text S2 for effects of rehearsal on rs-fcMRI). We did not find any significant difference in head movements estimated by realignment parameters among the three REST measurements (see Supplementary Text S3).

### Whole brain analysis of rs-fcMRI

We first conducted a whole-brain and hypothesis-free analysis of the degree of connectivity (Buckner et al., 2009; Hampson et al., 2012; Scheinost et al., 2012, 2013), which was defined for each voxel as the number of voxels to which the voxel was connected with a correlation coefficient *r* above or below a threshold. Preprocessing of rs-fcMRI and calculation of the degree of connectivity followed a previous study (Scheinost et al., 2012). Previous studies on positive connectivity used a threshold of *r* > 0.25 (Buckner et al., 2009; Hampson et al., 2012). These studies examined functional connectivities with positive correlations and set a positive threshold value (*r* > 0.25). By contrast, we aimed to change normally negative correlation toward zero or positive correlation. Therefore, we changed the sign of the threshold and the direction of the inequality sign (*r* < −0.25) to investigate regions where negative correlation changed toward zero or positive values. We examined statistically significant decreases in degree of connectivity between pre-REST and post-REST. Let us illustrate the relationship between the change of degree of connectivity and the change of correlation between lM1 and lLP when the correlation between two voxels in lM1 and lLP changed from −0.4 to 0.3 by the neurofeedback training. This connection is counted with the threshold of *r* < −0.25 (−0.4 < −0.25) before neurofeedback training, but it is not counted after training (0.3 > −0.25), and thus the degrees of connectivity of lM1 and lLP decrease by 1. If there exist 300 voxel pairs between lM1 and lLP whose correlations increase across the threshold −0.25 (e.g., from −0.3 to 0.1, from −0.4 to −0.2), then the degree of connectivity of lM1 and lLP decrease by 300. In summary, if the change in the degree of connectivity is a decrease, the result is in accordance with our prediction that a negative correlation between lM1 and lLP increases (e.g., from −0.4 to −0.2 or 0.1). First, we applied a threshold for *P* < 0.05 corrected for multiple comparisons at cluster level to the connectivity map, as well as a liberal threshold of *P* < 0.005 without correction, averaged across subjects separately for the three groups.

### ROI-based network analysis of rs-fcMRI

As we noted in the Introduction section, we adopted the definition of intrinsic networks in Laird et al. (2011) and investigated the changes in correlations between the MVN and DMN. Our network analysis was based on the correlation between ROIs from the two networks (Figure 4A and Table 1), including the target ROIs (M1 and LP). We included the SMA, which is related to a motor-imagery task (Grezes and Decety, 2001), in the ROIs of the MVN. Other regions in the MVN and DMN were determined according to a previous study on brain networks (Biswal et al., 2010). This study examined reproducibility of networks in rs-fcMRI across 1414 volunteers collected at 35 centers. One of the examined features was a negative correlation between the DMN and a group of regions named “task-positive” network (Fox et al., 2005) in a ROI-based correlation analysis. The ROIs for the DMN were located in the lateral parietal region (LP), the posterior cingulate cortex (PCC) and the medial prefrontal cortex (MPFC). We used these ROIs for the DMN. The ROIs for the task-positive network were located in the intra-parietal sulcus (IPS), the frontal eye field (FEF), and the middle temporal region (MT) (see Supporting Information of the study Biswal et al., 2010). We used the IPS- and FEF-ROIs since they are included in the MVN. As control networks besides the MVN and DMN, we defined ROIs in the visual and audition/speech networks. Because the previous study (Biswal et al., 2010) determined ROIs as spherical regions whose center was defined in MMN coordinates (Table 1), we followed these definitions. The other regions were anatomically defined according to the Brodmann area map in PickAtlas.

Concretely, data were analyzed using SPM8. The first two volumes of images in each run were discarded to allow for T1 equilibration. Functional images were temporally realigned to correct for the sequence of slice acquisition and then spatially aligned to the first remaining volume in a run with a six-parameter rigid-body transformation. Data were smoothed spatially with a Gaussian kernel of 6 mm full-width at half-maximum. To remove several sources of spurious variance unlikely to reflect spatially specific functional correlations, we applied regression analysis to the extracted time courses using explanatory variables: six realignment parameters, averaged signal over gray matter, white matter, and cerebrospinal fluid. We averaged the resultant residual time courses within each ROI. After these preprocessing steps, we calculated Fisher's *z*-transformed Pearson correlation coefficients (*r*) between the averaged time courses and then produced correlation matrices of all ROI pairs (Figure 4B).

### Bootstrapping method for analysis of network correlation

To investigate changes across subjects in correlations between networks, we adopted the bootstrapping method used in previous studies of resting-state fMRI (Efron and Tibshirani, 1986; Bellec et al., 2010) and analyzed the effect of training and its influence on combinations of networks under rest. The z-transformed correlation value during pre-REST was first subtracted from the value during post-REST for each region pair. We subtracted Fisher's z-transformed correlation matrix values before the training (pre-REST) from that immediately (Figure 5A: post-REST) or 2 months (Figure 5B: REST after 2 months) after the training of individual subjects and calculated within-group averages. To examine the differences in the training effect among six types of network pairs (MVN-MVN, MVN-DMN, DMN-DMN, MVN-control, DMN-control, and control-control), we divided the subtracted and then averaged correlation matrix into six network pairs corresponding to the types, and then we investigated which network pairs exhibited a significant number of region pairs in which the correlation values increased by more than the summation of mean and standard deviation across all region pairs and subject groups (colored cells in Figures 5A,B). Specifically, we computed confidence intervals (CI) for the number of colored cells for each network pair using a bootstrapping technique, as follows. We created 3000 resampled matrices by randomly sampling cells in the three matrices corresponding to the three subject groups, each of which was obtained by averaging the subtracted matrices within the subject group. Based on these matrices, we estimated the distribution of the numbers of colored cells for each network pair and calculated the (100 − α)% CI corresponding to a significance level at α (two-sided). To address the problem of multiple comparisons using Bonferroni correction, the upper range of the CI was raised from (100 − α)% to (100 − α/(6 × 3))% while taking into account the number of comparisons (i.e., number of network pairs × subject groups = 6 × 3). We determined which network pairs exhibited a larger number of colored cells than the corrected (100 − α)% CI.

## Results

### Changes in score during training

**Figure 2:**
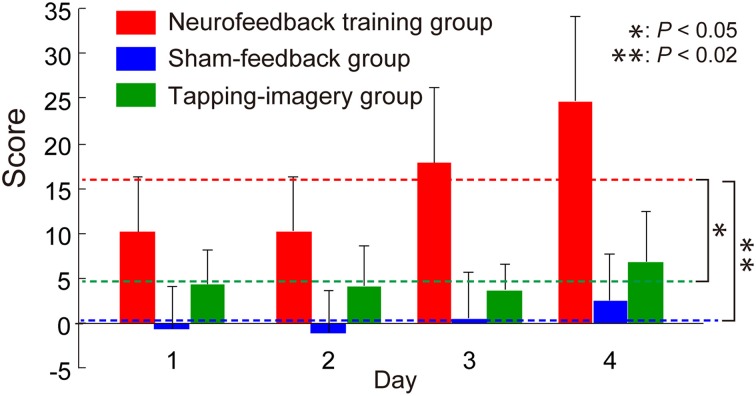
**Change in feedback score during neurofeedback training**. Neurofeedback scores averaged across subjects as a function of training days (error bars: standard errors). Broken lines indicate score values averaged across days for subject groups. Right asterisks indicate results of *post-hoc t*-tests on the averaged scores (see main text). *P*-values were corrected using the Bonferroni method.

Figure 2 shows the change in feedback score, representing the increment of the correlation, averaged across blocks and subjects as a function of training day. For comparison, we calculated the score from individual BOLD signals for the two control groups, although this score was not presented to the control groups. We applied a One-Way ANOVA to scores averaged across subjects separately for each day and found a significant effect of group [*F*_(2, 9)_ = 13.8, *P* = 0.0018]. *Post-hoc* *t*-tests indicated that the score of the neurofeedback training group is significantly higher than that of the sham-feedback group [*t*_(6)_ = 4.25, *P* = 0.0054; *P* < 0.02 after Bonferroni correction for two comparisons] or the tapping-imagery group [*t*_(6)_ = 3.04, *P* = 0.023; *P* < 0.05 after correction]. Note that the score represents the increment in the correlation compared to the initial block on day 1. Consequently, these results indicate that only the neurofeedback-training group exhibited a training effect averaged across the days, thus successfully increasing the correlation between the two areas by neurofeedback training.

### Changes in whole-brain connectivity

**Figure 3:**
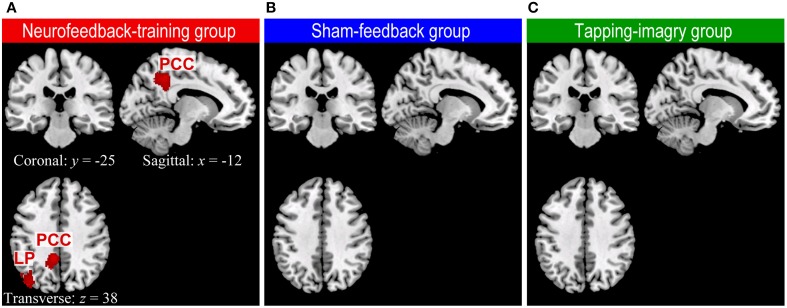
**Regions of the brain where degree of negative connectivity decreased from pre-REST to post-REST in neurofeedback training (A), sham-feedback (B), and tapping-imagery (C) groups**. We followed the methods of previous studies (Scheinost et al., 2012, 2013) for preprocessing of rs-fcMRI and computing degree of connectivity maps (Scheinost et al., 2012, 2013) except that we counted for each voxel the number of voxels to which it was correlated with *r* < −0.25 (see text). The difference in degree of connectivity between pre-REST and post-REST was examined voxel-by-voxel by using a paired *t*-test separately for the three subject groups. Regions with a significant decrease in degree of connectivity (red blobs) were identified by a cluster larger than 186 voxels (*P* < 0.05 corrected for multiple comparisons across the gray matter). Such regions were found only in the neurofeedback-training group. We used the AlphaSim program in Analysis of Functional Neuroimages (AFNI: http://afni.nimh.nih.gov/afni) for calculation of the cluster-level threshold. Note that functional images were smoothed spatially with a Gaussian kernel of 10 mm full-width at half-maximum in the preprocessing. *x, y*, and *z* indicate slice levels in NMI coordinates. LP, Lateral parietal region; PPC, Posterior parietal cortex.

Our whole-brain connectivity analysis found clusters with significant decreases in degree of connectivity only in the neurofeedback group but no clusters in the two control groups (Figure 3: *P* < 0.05 corrected for multiple comparisons at cluster level [cluster size > 186 voxels]). These clusters were found in the lLP and the posterior cingulate cortex (PCC). A decrease in the degree of connectivity means a decrease in the number of voxels to which each voxel in these clusters was connected with highly negative correlation (*r* < −0.25, see Materials and Methods). See section Whole Brain Analysis of rs-fcMRI for a detailed explanation on the consistent relationships between a decrease in the degree of connectivity and an increase in correlation. For instance, the change in correlation from *r* = −0.3 before the training to −0.1 or 0.2 after the training decreases the degree of connectivity. Such change in correlation was in accordance with our objective of applying connectivity-neurofeedback, i.e., to increase the correlation from negative toward zero or positive correlation. Changes in the degree of connectivity were found only in the neurofeedback group, which was the only one among the three groups to show the effect of neurofeedback training; this suggests a causal link between neurofeedback training and rs-fcMRI changes. We examined the robustness of these results with respect to the threshold setting for correlation values: the clusters in LP and PCC were consistently found at threshold ranges of −0.3 ≤ *r* ≤ −0.1 only in the neurofeedback training group, while no significant cluster could be found at a threshold of *r* < −0.25 in either the sham-feedback or the tapping-imagery group (several clusters appeared at much lower threshold than −0.25 in the sham-feedback [a cluster at *r* < −0.2 and three clusters at *r* < −0.1] and tapping-imagery [no cluster at *r* < −0.2 and three clusters at *r* < −0.1] groups). We applied a quite liberal threshold of *P* < 0.005 without correction for multiple comparisons to the above data. The decrease in connectivity was observed in the left SMA, lM1, the posterior cingulate cortex (PCC), lLP, the auditory cortex and other areas (see Supplementary Figure S3). Clusters having more than five voxels are listed in Supplementary Table S1 for the three subject-groups.

### Training effect in intrinsic networks

**Figure 4:**
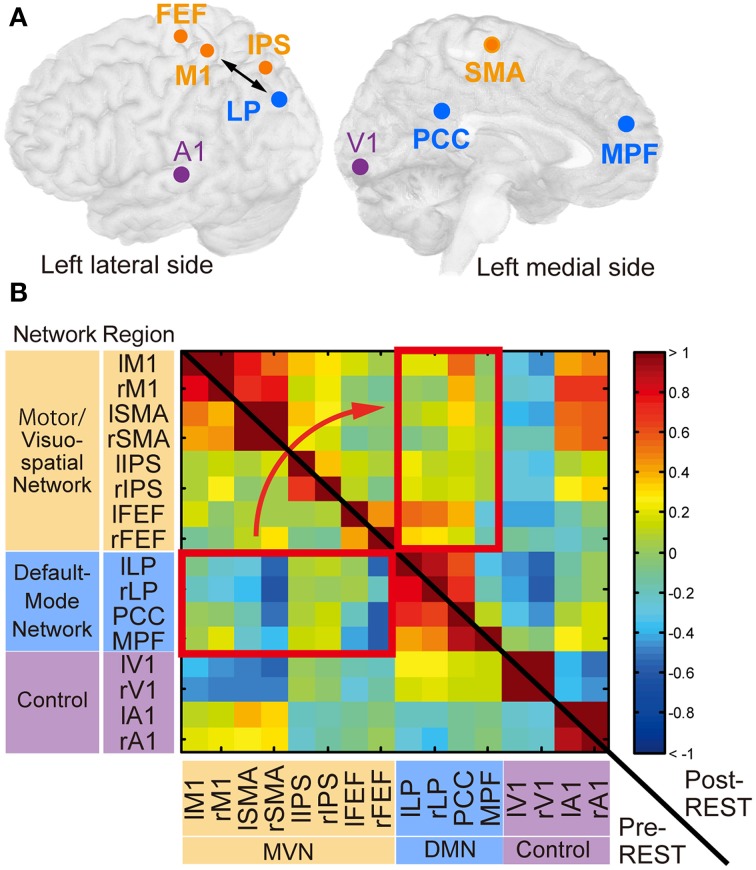
**Regions of interest (ROIs) and their functional connectivity. (A)** ROIs from which BOLD signal time courses were extracted. Circles indicate center positions of ROIs projected on the cortical surface. Orange, cyan and magenta circles correspond to ROIs in the motor/visuospatial network (MVN), the default mode network (DMN), and the control regions, respectively. The double-sided arrow indicates ROIs used for calculation of the feedback score in the neurofeedback training. This figure was created with multi_color software (http://www.cns.atr.jp/multi_color). **(B**) The z-transformed correlation matrix of all ROI pairs from a subject in the neurofeedback training group before (left-lower triangle area) and immediately after (right-upper rectangle area) training. Color bar indicates z-transformed correlation value. Red rectangles indicate region pairs between MVN and DMN. r, right; l, left. See Table 1 for abbreviations of region names.

We conducted a ROI-based correlation analysis between the MVN and DMN using 16 ROIs (Figure 4A and Table 1). Note that these ROIs were not specifically located at regions where a significant change in degree of connectivity was identified in the neurofeedback-training group but were the target ROIs (M1 and LP), regions related to motor-imagery (SMA), or those from previous studies on brain networks (see Materials and Methods). We calculated, separately for each of the 16 ROIs, the ratio of the number of voxels at which the degree of connectivity significantly changed (*P* < 0.005 uncorrected in group-level statistics, see above) across subjects to the number of voxels included in the ROI used for the network analysis. Ratios of significant-voxels averaged across 16 ROIs were 0.8% (SD: 2.1) for the neurofeedback-training group, 1.3% (4.8) for the sham-feedback group, and 0.6% (1.8) for the tapping-imagery group. We applied a One-Way ANOVA to the ratios but could not identify a significant effect of subject group [*F*_(2, 45)_ = 0.23, *P* = 0.79]. Thus, the results of the degree-of-connectivity analysis did not bias our selection of 16 ROIs for a particular group.

The temporal correlations between BOLD signal time courses averaged within each ROI were computed. Figure 4B shows z-transformed correlation matrices calculated from a representative subject in the neurofeedback-training group. The cell color indicates the z-transformed correlation value in each pair of regions. Before the training (pre-REST; lower-left triangle area), negative values (blue) were found in many region pairs between MVN and DMN (red rectangle), confirming the negative correlation. However, these values increased immediately after training (post-REST; upper-right triangle area; yellow/red).

**Figure 5:**
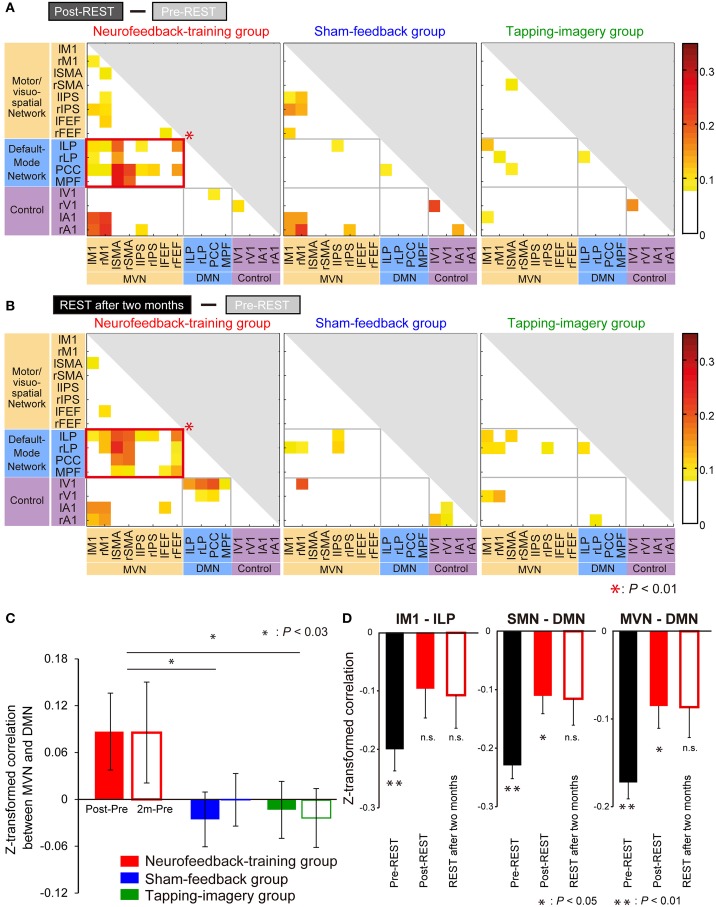
**Increase in correlation from pre- to post-training. (A)** Increase from pre-REST to post-REST. Yellow/red cells indicate ROI pairs whose correlation markedly increased (i.e., the increase was greater than the mean + SD [−0.02 + 0.09] across all pairs and groups). A red rectangle indicates a network pair having a significantly larger number of yellow/red cells than that generated by a random process according to a bootstrap sampling approach (*P* = 0.0001; *P* < 0.01 after the Bonferroni correction). **(B)** Increase from pre-REST to “REST after 2 months.” Colored cells indicate that the increase is greater than the mean + SD (0.00 + 0.07). Color bars indicate increments from pre- to post-training. **(C)** Increase in correlation averaged across subjects and region pairs between the MVN and DMN (error bars: standard errors). Filled bars indicate the increase from pre-REST to post-REST, and open bars indicate the increase from pre-REST to REST after 2 months. Asterisks indicate results of *post-hoc* comparisons on the averaged increase (see main text). **(D)** Z-transformed correlation in pre- and post-training for the neurofeedback training group. BOLD signal time courses were averaged within individual ROIs: lM1, lLP, sensorimotor network (SMN) and DMN. Correlations were calculated using the averaged time courses between lM1 and lLP (left), SMN and DMN (middle), and MVN and DMN (right). They were averaged across subjects during the three resting state activity measurements (error bars: standard errors). Asterisks indicate the results of one-sample *t*-tests that examined whether the z-transformed correlation is significantly smaller than zero after *P*-values were corrected for three comparisons using the Bonferroni method (see main text).

To investigate consistent changes across subjects, we first subtracted the z-transformed correlation value during pre-REST from the value during post-REST for each region pair. The colors in Figure 5A indicate the subtracted values averaged across subjects separately for the groups. To indicate pairs of regions in which correlation markedly increased, cells (region pairs) in Figure 5A are colored if their values are higher than the summation of mean value and standard deviation (mean + SD: −0.02 + 0.09) across all region pairs and subject groups. We examined which network pairs in the correlation matrix exhibited a significantly larger number of colored cells (compared to the number generated by a random process) when the matrix was partitioned into six areas according to the type of network combination (MVN-MVN, MVN-DMN, DMN-DMN, MVN-control, DMN-control, and control-control; gray or red border lines in Figure 5). The bootstrap sampling approach (see Materials and Methods for bootstrapping method) was used to estimate the probability of the number of colored cells, if they were generated by a random process, while it took into account the total number of cells (region pairs) in each network pair. The analysis revealed that only the network pair of the MVN and DMN in the neurofeedback-training group exhibited a significant number of colored cells (*P* = 0.0003, computed using bootstrap samples; *P* < 0.01 corrected by the Bonferroni method across six network pairs and three experimental groups). This finding suggests that the neurofeedback training significantly increased the correlation in intrinsic activity only between the MVN and DMN, and only for the neurofeedback-training group. Note that 16 ROIs are small representations of the brain's functional networks, so to verify our conclusions we increased the number of ROIs from 16 to 33 to cover a broader range of regions in the cerebral cortex and found similar results to those in Figure 5 (see Supplementary Table S2 and Supplementary Figure S5A).

Furthermore, highly similar patterns were found in matrices obtained by the subtraction of correlations in pre-REST from those in REST after 2 months (Figure 5B), suggesting that the training effect was stably maintained for 2 months. It should be noted that the increase in correlation value was not restricted to the region pair used for neurofeedback (lM1—lLP) but was also found in the other region pairs between the MVN and DMN. In an additional, more extensive network analysis, we applied the same analyses to a larger number of ROIs and obtained similar results (Supplementary Figure S5B).

Regarding the control groups, no significant increase in correlation for any combination of network types was found, either in post-REST (middle and right panels of Figure 5A) or REST after 2 months (those of Figure 5B). Consequently, a significant increase in correlation between the networks was found only in the neurofeedback-training group.

### Changes in correlation-values

Figure 5C indicates an increase in the z-transformed correlation from pre- to post-training between the MVN and DMN, which were averaged across subjects and shown separately for the three subject groups. A Two-Way ANOVA (subject group x measurement [Post—Pre or 2 m—Pre]) identified a significant effect of subject group [*F*_(2, 60)_ = 5.32, *P* = 0.007] but no significant effect of measurement [*F*_(1, 60)_ = 0.02, *P* = 0.88] or interaction [*F*_(2, 60)_ = 0.12, *P* = 0.89]. This suggests significant differences in the increase across the groups. *Post-hoc* comparisons found a significant difference between neurofeedback-training and sham-feedback groups (*P* = 0.011; *P* < 0.03 after correction for two comparisons), and neurofeedback-training vs. tapping-imagery (*P* = 0.010; *P* < 0.03 after correction). However, there was no significant difference in sham-feedback and tapping-imagery (*P* = 1.00, n.s.). We applied a One-Way ANOVA to correlations between M1 and LP during the pre-REST (i.e., before neurofeedback training) across the three subject groups but did not find a significant difference [*F*_(2, 30)_ = 2.39, *P* = 0.11, n.s.]. Because of non-significant difference in correlation before the training, the significant difference in the correlation increase found across the three groups was caused by the difference in the training conditions.

The above subtraction of correlation values in the pre-training from those in the post-training indicated a significant increase in correlation between the MVN and DMN in the neurofeedback group. To investigate changes in correlation at different levels of regional sizes, we further calculated correlation values by changing pairs of ROIs and networks: lM1—lLP, sensorimotor network (Laird et al., 2011) (SMN including M1)—DMN, and VMN—DMN. We applied a Three-Way ANOVA (group × measurement [Pre, Post or 2 m] × ROI pair) to the z-transformed correlation values before subtraction, and found a significant effect of the group [*F*_(2, 270)_ = 3.59, *P* = 0.029]. Therefore, we applied Two-Way ANOVA (measurement × ROI pair) to the correlation values separately for the three groups, and found a significant effect of measurement only for the neurofeedback-training group [*F*_(2, 99)_ = 6.56, *P* = 0.002; *P* < 0.01 after correction for three comparisons, see Supplementary Table S3 for other effects and groups].

Figure 5D shows correlation values averaged across subjects of the neurofeedback-training group separately for pairs of ROIs. We applied one-sample *t*-tests to examine whether z-transformed correlation is significantly smaller than zero. Consequently, for the lM1-lLP connectivity (left panel), the correlation was significantly negative in the pre-REST (*t*_(11)_ = −5.18, *P* < 0.001 [*P* < 0.01 corrected for three comparisons]) but not in the post-REST (*t*_(11)_ = −1.89, *P* = 0.26 [n.s.]) or the REST after 2 months (*t*_(11)_ = −1.81, *P* = 0.29 [n.s.]). For the SMN-DMN connectivity (middle), the correlation was significantly negative in the pre-REST (*t*_(11)_ = −9.73, *P* < 0.001 [*P* < 0.01]) and the post-REST (*t*_(11)_ = −3.47, *P* = 0.02 [*P* < 0.05]) but not in the REST after 2 months (*t*_(11)_ = −2.45, *P* = 0.10 [n.s.]). For the MVN-DMN connectivity (right), the correlation was significantly negative in the pre-REST (*t*_(11)_ = −8.83, *P* < 0.001 [*P* < 0.01]) and the post-REST (*t*_(11)_ = −3.26, *P* = 0.02 [*P* < 0.05]) but not in the REST after 2 months (*t*_(11)_ = −2.40, *P* = 0.11 [n.s.]). Therefore, the negative correlation in the pre-REST consistently increased from negative toward zero after the neurofeedback training for all three ROI and network pairs.

## Discussion

We hypothesized that positively correlated activations of two regions evoked by repeated experiences induces a long-term increase in their functional connectivity, which is the temporal correlation values within intrinsic networks. To directly examine this hypothesis, we induced an experimentally traceable change in the correlation between specific networks, the MVN and DMN, by evoking correlated activation between two specific regions based on the connectivity-neurofeedback training (Fukuda et al., 2011). The training led to a significant increase in correlation between the two networks under rest, and this increase was preserved for more than 2 months. Consistent and significant changes in the feedback score, changes in degree of connectivity from pre- to post-training, and changes in network connectivity revealed by the ROI-based analysis from pre- to post-training were all identified only in the neurofeedback-training group. This suggests a causal link between neurofeedback training and rs-fcMRI changes.

### Instruction to subjects

We instructed subjects to perform a mental imagery task during the training session. This instruction, however, is unlikely directly related to modulation of temporal correlation between lM1 and lLP because a meta-analysis of more than 3000 experiments indicated that lM1 and lLP do not co-activate under known tasks including motor imagery tasks (Toro et al., 2008). There is no task strategy known to be efficient for increasing correlation between M1 and LP, so we chose the reinforcement-leaning method, in which the desired response is reinforced by reward during a trial-and-error search without an effective instruction of strategies. This method has been adopted in previous fMRI neurofeedback experiments (Bray et al., 2007; Shibata et al., 2011). However, the motor imagery should have certainly contributed to consistent temporal changes in BOLD time courses in M1 (deCharms et al., 2004; Bray et al., 2007) as well as in LP at the initial stage of learning.

### Control groups and decrease in network correlation

There was no significant change in score during training or rs-fcMRI in the sham-feedback group. This indicates that the spurious increase in score/reward, which does not correctly reflect the actual correlation between the two ROIs in one's own brain, even combined with mental motor imagery, cannot induce increases in correlation between the ROIs or changes in the intrinsic network. No significant change in the tapping-imagery group suggests that the repetition of tapping imagery cannot change correlation between the ROIs or the network.

We could not identify a consistent decrease from pre-REST to either post-REST or REST after 2 months (see Supplementary Figures S4, S6). This suggests that the long-lasting increase was not due to daily fluctuations in rs-fcMRI but to the effect of the connectivity-neurofeedback training.

### Change in functional connectivity during neurofeedback training

We used a neurofeedback training method to induce co-modulation between two specific regions. However, these two regions also exhibit connectivity with many other regions. Therefore, many regions may have been simultaneously modulated, and the correlations among various regions may have increased simultaneously during training. To examine this possibility, we investigated the change in correlated activity during neurofeedback training in regions other than the lM1 and lLP.

Supplementary Figure S7 shows changes in patterns of correlation during the imagery period from day 1 to day 4 following the conventions of Figure 5. The bootstrap sampling method (see legend of Supplementary Figure S7 for details) revealed that only the network pair of MVN and DMN contained a significant number of colored cells (*P* = 0.0017; *P* < 0.05, corrected by Bonferroni method across six network pairs). This suggests that a significant increase in correlation was restricted to the network pair in which the correlation was manipulated. Moreover, the most prominent increase was found in region pairs between motor regions (M1 and SMA) and the DMN (blue rectangle in Supplementary Figure S7). No significant increment in correlation was found for any network combination in either of the control groups. Thus, an effect of experimental manipulation during training on connectivity was found only in the neurofeedback training group, and this effect was restricted to the correlation between the MVN and DMN, most prominently in the correlation between motor regions and the DMN.

Correlations among BOLD-signal time series are known to be vulnerable to the artifact, such as scanner-dependent drift and head movement (Power et al., 2012). We applied motion realignment and temporal filtering but could not remove motion effect explicitly during online feedback computation (e.g., correct signal using realign parameters). Thus, we cannot completely reject such an artifact during training in the current design. However, we confirmed that the functional connectivity changes induced by training in the independent resting-state data remained while head movement effects were removed, suggesting changes in functional connectivity is not a mere reflection of a movement-related artifact. It is critical to improve online motion correction algorithms and artifact removal techniques (e.g., Zaitsev et al., 2006; Koush et al., 2012) for future studies.

### Increase in negative correlation

Our results do not exclude the possibility that changes in other regions may mediate or facilitate changes in correlation between target regions because we calculated correlation in BOLD time courses between two regions during the training. Furthermore, there should be a number of pathways between the two regions. These complicate the relationships between “correlation” and “connectivity,” especially for the change in negative correlation. When the correlation increases for example from *r* = −0.5 to *r* = −0.1, (1) the strength of inhibitory connection may be weakened at some connections, and/or (2) an excitatory connection may emerge and increase at other connections. The current rs-fcMRI method cannot discriminate these different neural mechanisms, and in the future we need to utilize higher temporal resolution techniques and/or depend on animal models.

### Extension of fMRI-based neurofeedback therapeutics

Analyses of rs-fcMRI in large-scale brain networks have found abnormality with many psychiatric and neurological disorders (for reviews: Fox and Raichle, 2007; Broyd et al., 2009). Specific abnormal connectivity between limited brain regions has been identified in many psychiatric disorders, for instance, the connectivity between orbitofrontal cortex and ventral striatum in obsessive-compulsive disorder (OCD) (Harrison et al., 2009) and connectivity between left dorsolateral prefrontal cortex and subgenual cingulate in major depressive disorder (Fox et al., 2012). Many clinical studies have demonstrated that various types of treatments normalized pathological functional connectivity. Such treatments include pharmacotherapy [depressive disorder (Anand et al., 2005), schizophrenia (Abbott et al., 2013a), attention deficit/hyperactivity disorder (Wong and Stevens, 2012), and Alzheimer's disease (Goveas et al., 2011)], repetitive transcranial magnetic stimulation (depressive disorder, Liston et al., 2014), electroconvulsive therapy (depressive disorder, Perrin et al., 2012), deep brain stimulation (OCD, Figee et al., 2013), and ROI-based fMRI neurofeedback training (OCD, Scheinost et al., 2013). Moreover, some of these studies indicate that the magnitude of change in connectivity is significantly correlated with curing effect (Figee et al., 2013; Scheinost et al., 2013; Liston et al., 2014). FMRI neurofeedback equipped with high spatial resolution has the potential for direct normalization of the functional connectivity between specific regions and may provide entirely new therapeutic methods for psychiatric disorders. Recently, fMRI neurofeedback training (Scheinost et al., 2013) on regulation of activity in a single ROI in the orbitofrontal cortex, for subclinical contamination anxiety, was found to normalize the functional connectivity of that ROI. Furthermore, changes in resting-state global connectivity of the orbitofrontal ROI measured by degree of connectivity were correlated with the alleviation of anxiety. However, no existing treatment, including fMRI neurofeedback, can selectively change specified functional connectivity between two selected brain areas, which is essential for selective and effective therapeutic treatment of psychiatric disorders. Here, as a proof of concept, we demonstrated that fMRI “connectivity” neurofeedback training could change functional connectivity, as revealed by a correlation in rs-fcMRI between two selected regions, and that the effect of training could be preserved for a long time, which is crucial for clinical applications.

DMN has been frequently found in aberrant connectivity in many psychiatric disorders (for reviews: Fox and Raichle, 2007; Broyd et al., 2009). It has been shown that the negative correlation between parts of DMN and the dorsolateral prefrontal cortex is stronger in patients with major depressive disorder than healthy controls, and that electroconvulsive therapy reduces this correlation (Perrin et al., 2012; Abbott et al., 2013b). Our method can be applied to therapeutics for major depressive disorder by weakening the negative correlation. By contrast, the negative correlation between the DMN and regions in the task-positive network is known to have positive effects on cognitive functions: for instance, strength of the negative correlation is positively correlated with performance in working memory (Hampson et al., 2010; Keller et al., 2015) or small variability in reaction time in a conflict task (Kelly et al., 2008). Thus, weakening the negative correlation may cause negative effects on higher order cognitive functions. In our current study, the effect of neurofeedback training was found on correlation between the DMN and MVN but not between the DMN and the executive network (Supplementary Figure S5), which is the most likely related to higher order functions. However, when considering the long-term effects and potential ability to change connectivity determined by genetic and evolutional factors, as we mentioned in the introduction part, future studies need careful experimental deigns from an ethical perspective if the connectivity includes the DMN regions. For instance, an experiment should include monitoring cognitive functions and behavioral variables, which could be influenced by the training, and the training should be terminated if aversive effects are identified.

### Conflict of interest statement

Japanese patent 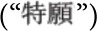 2013-259554 (pending) on the use of connectivity-neurofeedback. The authors declare that the research was conducted in the absence of any commercial or financial relationships that could be construed as a potential conflict of interest.
