# Supplementary material for: Functional MRI neurofeedback training on connectivity between two regions induces long-lasting changes in intrinsic functional network
Source: Front Hum Neurosci. 2015 Mar 30;9:160. doi: 10.3389/fnhum.2015.00160 (PMC4377493; doi:10.3389/fnhum.2015.00160)
Supplement: Supplementary file 1 [file Presentation1.PDF]

Supplementary Material

Functional MRI neurofeedback training on connectivity between two regions induces long-lasting changes in intrinsic functional network

Fukuda Megumi [1,2,3], *, Ayumu Yamashita [1,4], Mitsuo Kawato [1,2], and Hiroshi Imamizu [1,5]*

[1] Advanced Telecommunications Research Institutes International, 2-2-2 Hikaridai, Keihanna Science City, Kyoto 619-0288, Japan
[2] Nara Institute of Science and Technology, 8916-5 Takayama, Ikoma, Nara 630-0192 Japan
[3] Institute of Cognitive Neuroscience, University College London, 17 Queen Square, London, WC1N 3AR, UK
[4] Kyoto University, 36-1 Yoshida-Honmachi, Sakyo-ku, Kyoto 606-8501, Japan
[5] Center for Information and Neural Networks, National Institute of Information and Communications Technology and Osaka University, 1-4, Yamadaoka, Suita, Osaka 565-0871, Japan

* **Correspondence:** Hiroshi Imamizu, Cognitive Mechanisms Laboratories, Advanced Telecommunications Research Institutes International, 2-2-2 Hikaridai, Keihanna Science City, Kyoto 6190288, Japan (imamizu@gmail.com), or Fukuda Megumi, Institute of Cognitive Neuroscience, University College London, 17 Queen Square, London, WC1N 3AR, UK (megumi.fukuda.11@ucl.ac.uk)

1. Supplementary Data

1.1. Supplementary Text S1: Post-experiment debriefing

After the experimental procedure (after 'REST after two months'), subjects were asked 1) what they did during the training, 2) what kind of mental imagery helped most to increase their score (in the neurofeedback and sham-feedback groups), 3) whether they noticed that simply performing tapping imagery was not enough to increase their score (in the neurofeedback and sham-feedback groups), and 4) whether they noticed that feedback was not calculated from their BOLD signal but from the signals of another person. Consequently, we confirmed that most subjects produced imagery related to motor function in the task, especially hand movement, and that almost all of the sham-feedback training subjects were unaware that the feedback scores did not reflect their own brain activity (11 out of 12). None of the subjects were aware of how the experiment was actually conducted.

1.2. Supplementary Text S2: Effects of rehearsal on rs-fcMRI

During the measurements of post-REST and 'REST after two months,' subjects were instructed not to recall what they did during neurofeedback training, but they might not have followed this instruction. However, if rs-fcMRI reflects correlational structures in the activity of a low-frequency component (Fox et al., 2007) (especially if it is less than 0.06 Hz in our data), it is unlikely that the subjects' recall affected changes in rs-fcMRI signals. This is because a 0.06 Hz high-pass filter was applied to the BOLD signal time series during neurofeedback training, and thus, neurofeedback training was effective only for frequency components higher than 0.06 Hz. To investigate the main component in rs-fcMRI, we estimated the power spectrum density of BOLD signals during REST

after two months. The density was estimated after preprocessing (see "ROI based network analysis of rs-fcMRI" in Materials and Methods), and it was averaged within ROIs and across subjects. As a result, the dominant frequency component was less than 0.05 Hz (Fig. S2). Therefore, it is unlikely that modulation of BOLD signals during the resting state occurred in the same frequency as that during the training, or that recall or rehearsal, if any, affected rs-fcMRI.

1.3. Supplementary Text S3: Head movements during resting-state activity measurements

Parameters obtained during realignment of functional-image volumes (see "ROI-based network analysis of rs-fcMRI" in Materials and Methods) were used to quantify head movements. We calculated variances of three parameters related to translational displacements along X, Y and Z axes, and averaged the variances across parameters within each subject separately for pre-REST, post-REST, and REST after two months. We applied a one-way ANOVA to the averaged variances but could not find a significant difference among the three RESTs ($F(2, 33) = 0.49$, $P = 0.62$). We also analyzed parameters related to rotational displacements of pitch, yaw and roll but could not find a significant difference among the RESTs ($F(2, 33) = 0.45$, $P = 0.64$). Therefore, it is unlikely that changes in head movements explained the changes in rs-fcMRI connectivity.

2. Supplementary Figures and Tables

2.1. Supplementary Table

Supplementary Table S1. Clusters found in degree of connectivity analysis

	Network	ICA	ROI	Neurofeedback Group	Sham-feedback Group	Tapping-imagery Group
	Emotion/ interception	1		#2(176)	#3(27)	#2(22), #3(78)
		2		#3(6)	#4(6), #6(6)	
		3				
		4		#3(6), #5(26), #14(11)	#5(9), #11(9)	
		5			#1(73), #2(17)	#1(6), #3(78)
	**Motor/ visuospatial**	6	FEF, SMA	#13(17), #14(11)	#9(110), #10(17)	
		7	IPS		#8(17), #10(17)	
		8	M1	#5(26), #11(98)		#9(7)
		9			#11(9)	#10(7)
	Visual	10			#7(55)	#6(18), #7(16)
		11				
		12	V1			
Divergent	**Default**	13	LP, PCC, MFP	#6(652), #10(283)	#4(6), #7(55)	#4(32), #8(15)
	Audition	14				
		15		#1(10), #5(26), #8(140), #12(6)		#8(15)
		16	A1	#4(45), #7(11)		#4(32), #5(71), #6(18), #7(16)
		17		#5(26), #7(11)	#5(9)	
		18		#9(18)		

Note: #Cluster ID (Number of voxels). Results were thresholded at $P < 0.005$ without correction for multiple comparisons. Clusters having more than five voxels are listed. Network names follow those of a previous study (Laird et al., 2011). The column labeled ICA indicates a number given to a network identified by an independent component analysis in the study (Laird et al., 2011). The column labeled ROI indicates labels of 16 ROIs as listed in Table 1 and Figure 4A. Clusters partially included in more-than-two ICA components (networks) are listed in each of the components: for instance, #7(11) in the neurofeedback-training group is listed in both 16th and 17th components.

Supplementary Table S2. Thirty-three regions of interest for analysis of resting state fMRI

Network	Label	Anatomical region	Area / MNI coordinates
Executive control network	dmPFC	Dorsal medial prefrontal cortex	(0, 24, 46)
	laPFC(1)	Left anterior prefrontal cortex	(-44, 45, 0)
	raPFC(1)	Right anterior prefrontal cortex	(44, 45, 0)
	lSP	Left superior parietal	(-50, -51, 45)
	rSP	Right superior parietal	(50, -51, 45)
Salience network	DAC	Dorsal anterior cingulate	(0, 21, 36)
	laPFC(2)	Left anterior prefrontal cortex	(-35, 45, 30)
	raPFC(2)	Right anterior prefrontal cortex	(32, 45, 30)
	lINS	Left insula	(-41, 3, 6)
	rINS	Right insula	(41, 3, 6)
	lLP(2)	Left lateral parietal	(-62, -45, 30)
	rLP(2)	Right lateral parietal	(62, -45, 30)
Sensorimotor network	lM1*	Left motor cortex	Area 4
	rM1*	Right motor cortex	Area 4
	lSMA*	Left supplementary motor area	Area 6
	rSMA*	Right supplementary motor area	Area 6
Dorsal attention network	lFEF*	Left frontal eye field	(-25, -13, 50)
	rFEF*	Right frontal eye field	(25, -13, 50)
	lpIPS*	Left posterior intra-parietal sulcus	(-25, -57, 46)
	rpIPS*	Right posterior intra-parietal sulcus	(25, -57, 46)
	laIPS	Left anterior intra-parietal sulcus	(-44, -39, 45)
	raIPS	Right anterior intra-parietal sulcus	(41, -39, 45)
Default-mode network	lLP(1)*	Left lateral parietal	(-45, -67, 36)
	rLP(1)*	Right lateral parietal	(45, -67, 36)
	MPF*	Medial prefrontal	(-1, 47, -4)
	PCC*	Posterior cingulate cortex/precuneus	(-5, -49, 40)
	MDT	Medial dorsal thalamus	(0, -12, 9)
	lIFT	Left inferior temporal	(-61, -24, -9)
	rIFT	Right inferior temporal	(58, -24, -9)
Control (vision and auditory networks)	lV1*	Left primary visual cortex	Area 17
	rV1*	Right primary visual cortex	Area 17
	lA1*	Left auditory cortex	Areas 41 & 42
	rA1*	Right auditory cortex	Areas 41 & 42

Note: ROIs with asterisks are the same as those used in the previous analysis (Table 1).

Supplementary Table S3. Results of two-way ANOVA

Effect	*F*-value	Uncorrected *P*-value	*P*-value corrected for three comparisons
Neurofeedback training group			
Measurement	$F(2, 99) = 6.56$	0.002	< 0.01
ROI pair	$F(2, 99) = 0.68$	0.508	n.s.
Measurement x ROI pair	$F(4, 99) = 0.05$	0.994	n.s.
Sham-feedback group			
Measurement	$F(2, 99) = 0.65$	0.562	n.s.
ROI pair	$F(2, 99) = 3.11$	0.049	n.s.
Measurement x ROI pair	$F(4, 99) = 0.08$	0.989	n.s.
Tapping-imagery group			
Measurement	$F(2, 99) = 1.48$	0.235	n.s.
ROI pair	$F(2, 99) = 1.54$	0.222	n.s.
Measurement x ROI pair	$F(4, 99) = 2.66$	0.076	n.s.

2.2. Supplementary Figures

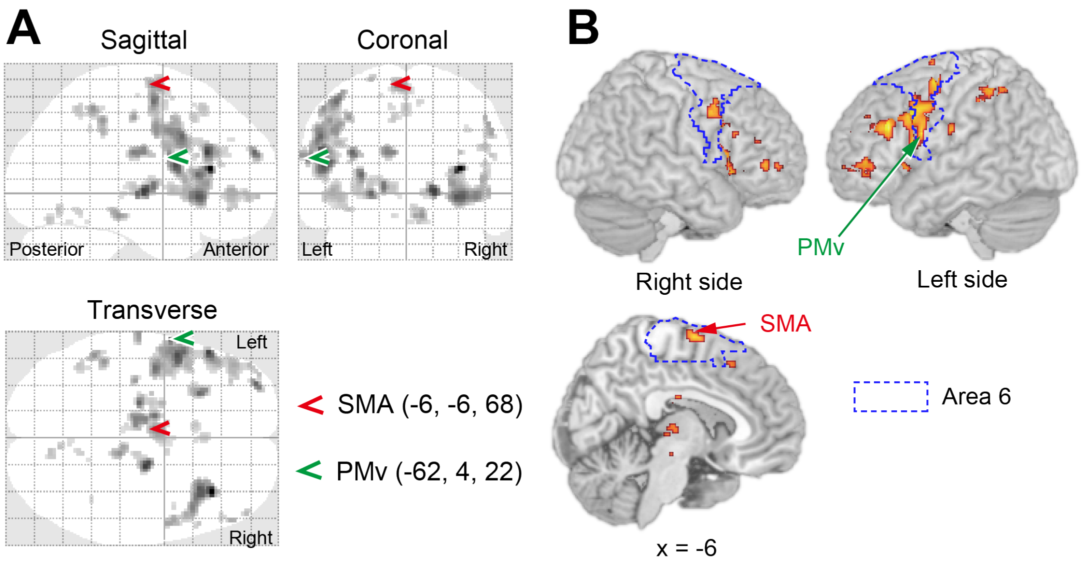

Supplementary Figure S1. Subtraction result of activity during resting-state measurements from activity during imagery periods of neurofeedback training. Resting-state activity was averaged across pre-REST, post-REST, and REST after two months and subtracted from activity during imagery periods of neurofeedback training to investigate the difference in activity between the tapping imagery and resting-state measurement. (**A**) Group analysis of subjects in the neurofeedback training group (n = 12). Blobs indicate subtracted activity that are thresholded at $P < 0.001$ uncorrected for multiple comparisons ($t(11) > 4.0$). Cursors (<) indicate positions of activity peaks in MNI coordinates of clusters that survived $P < 0.05$ corrected within Area 6 (regions enclosed by blue curves in Fig. S1B), where many studies have reported activity during mental motor imagery tasks (Grezes et al., 2001). SMA: supplementary motor area, PMv: ventral premotor region. (**B**) Activity projected to the right or left surfaces of the brain (top right or left, respectively), or activity rendered on the sagittal slice (bottom). Region of interest for Area 6 was determined according to the anatomical map in PickAtlas (http://fmri.wfubmc.edu/software/PickAtlas).

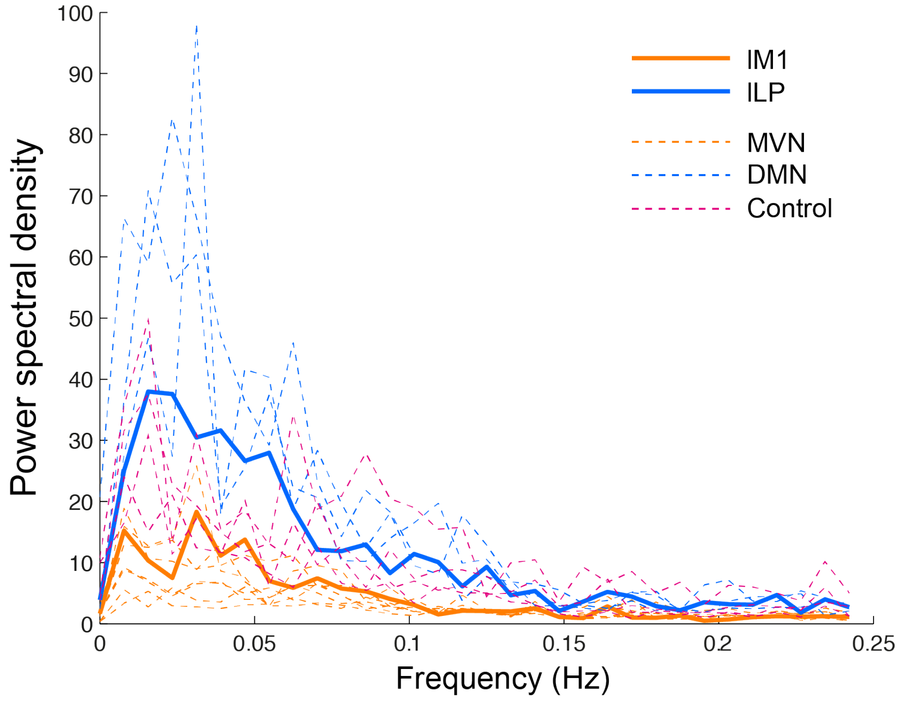

Supplementary Figure S2. Power spectrum density of BOLD signal time series during REST after two months. Spectrum density was estimated from a time series after preprocessing (see “ROI based network analysis of rs-fcMRI” in Materials and Methods) and averaged within each ROI. Thick lines indicate density profiles for lM1-ROI (orange) and lLP-ROI (cyan). Dashed lines indicate profiles for seven ROIs in MVN except for lM1 (orange), three ROIs in the DMN except for lLP (cyan), and four ROIs in the control regions (magenta). See Table 1 for definitions of ROIs. This figure indicates that the dominant component was less than 0.05 Hz in BOLD signals for rs-fcMRI. Because the 0.06 Hz high-pass filter was applied to the signal time series during the neurofeedback training, the training was effective only for frequency components higher than 0.06 Hz. Therefore, it is unlikely that the modulation of BOLD signals during the resting state occurred in the same frequency as that during the training, or that recall or rehearsal affected the rs-fcMRI. Any effect of subjects’ recall or rehearsal on rs-fcMRI was also refuted by the fact that the correlational structures of rs-fcMRI for the three rest conditions in the two control groups, as well as the pre-REST for the experimental group, were nearly identical (see Figs. 4 and 5). If recall or rehearsal were a cause of rs-fcMRI changes, we would have observed differences between these seven correlational structures.

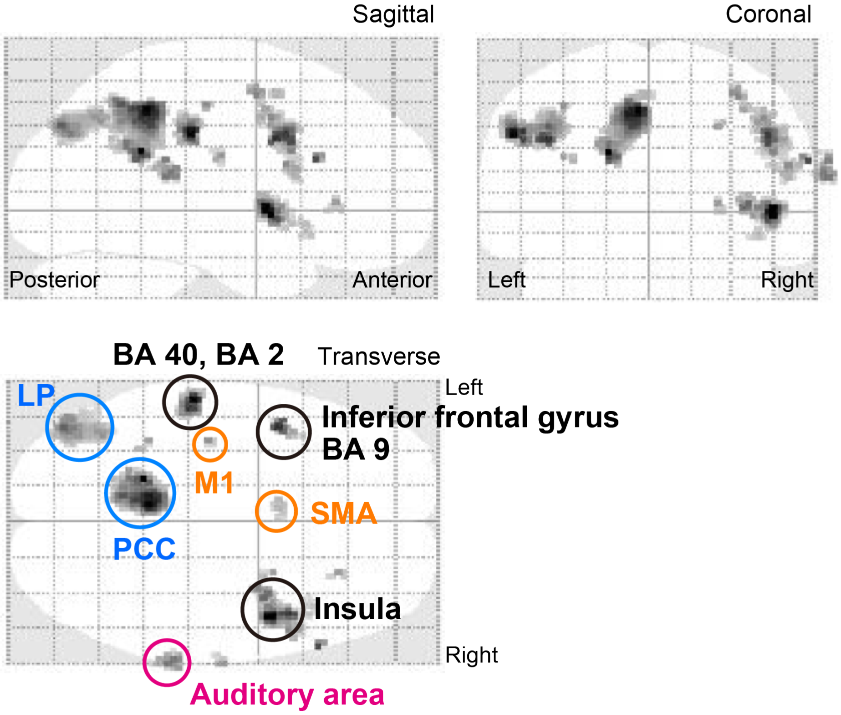

Supplementary Figure S3. Regions where degree of negative connectivity decreased from pre-REST to post-REST in neurofeedback training group at lower threshold than that in Figure 3 (main text). The same results as Figure 3A are projected to the sagittal, coronal and transverse planes but thresholded at $P < 0.005$ without correction for multiple comparisons. Circles indicate clusters of more than 5 voxels. Orange and cyan circles indicate regions belonging to the motor/visuospatial networks and the default mode network, respectively. BA: Brodmann area, M1: primary motor region, SMA: supplementary motor area, LP: lateral parietal region, PCC: posterior cingulate cortex.

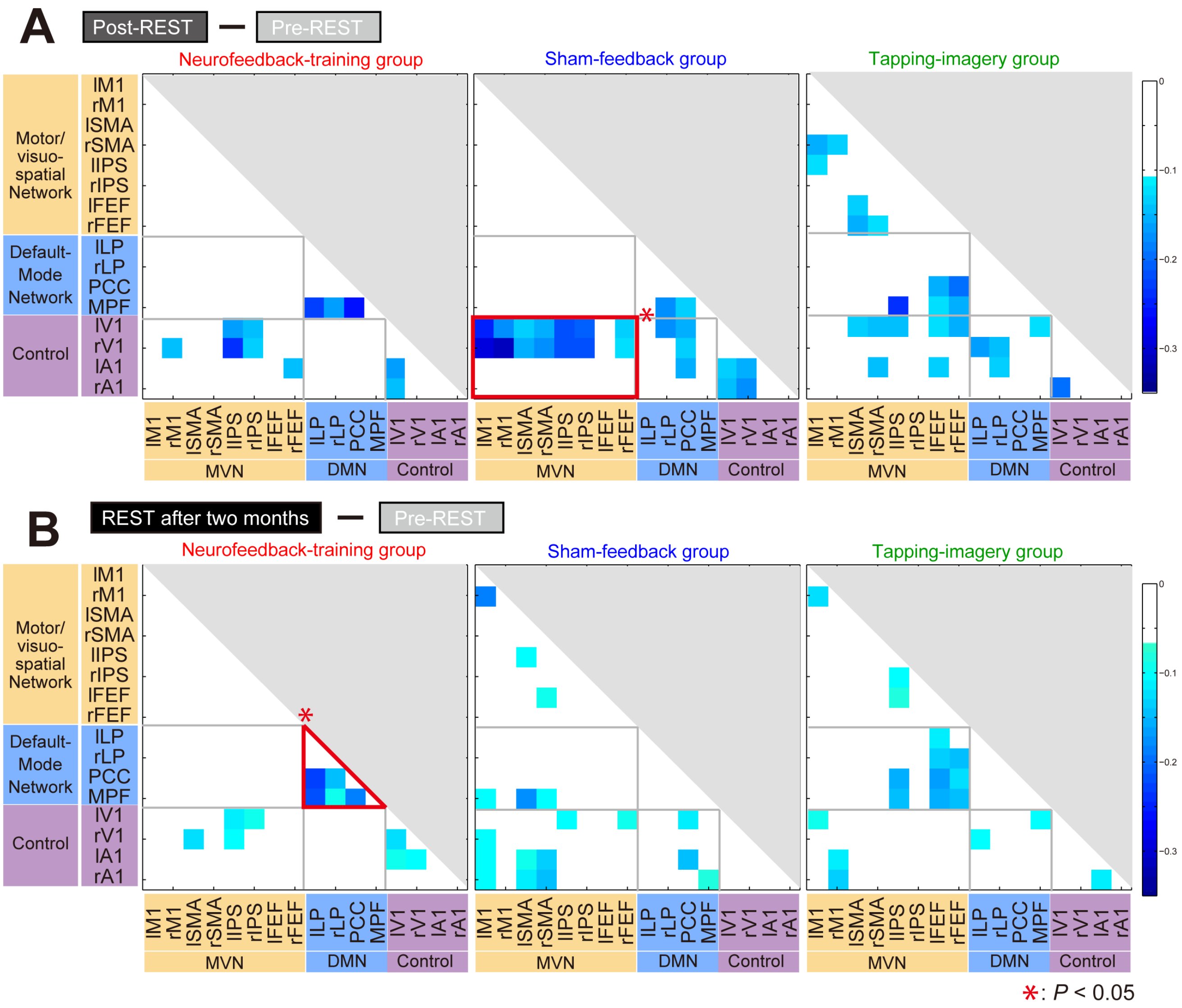

Supplementary Figure S4. Decrease in z-transformed correlation values from pre- to post-training. We used the same bootstrap approach as that for Figure 5 to test whether there was a significant decrease in correlation in any combination of network types. (**A**) Decrease in correlation from pre-REST to post-REST. Conventions follow those of Figure 5 except for cyan/blue cells, which indicate ROI pairs whose correlations markedly decreased (more than mean - SD: -0.02 - 0.09) from pre-REST to post-REST. The red rectangle indicates a network pair having a significantly larger numbers of colored cells than that generated by a random process according to a bootstrap sampling approach (decrease in MVN-Control in sham-feedback group, P = 0.0003; P < 0.05 after Bonferroni correction; see Materials and Methods). (**B**) Decrease in correlation from pre-REST to REST after two months. Cyan/blue cells indicate ROI pairs in which correlations markedly decreased (more than mean - SD: 0.00 - 0.07) from pre-REST to REST after two months. The red rectangle indicates a network pair having a significantly larger numbers of colored cells than that generated by a random process according to a bootstrap sampling approach (decrease within DMN in neurofeedback training group, P = 0.0011; P < 0.05 after Bonferroni correction). Therefore, we did not observe a consistent decrease pattern in the two comparisons (Pre-REST and Post-REST, Pre-REST and REST after two months). When the number of ROIs was increased from 16 to 33 (Fig. S6), no combination of network pairs had a significant decrease from pre-REST to either post-REST or

REST after two months. These results suggest that the long-lasting increase in the neurofeedback training group was not due to daily fluctuations in rs-fcMRI but to the effect of the connectivity-neurofeedback training.

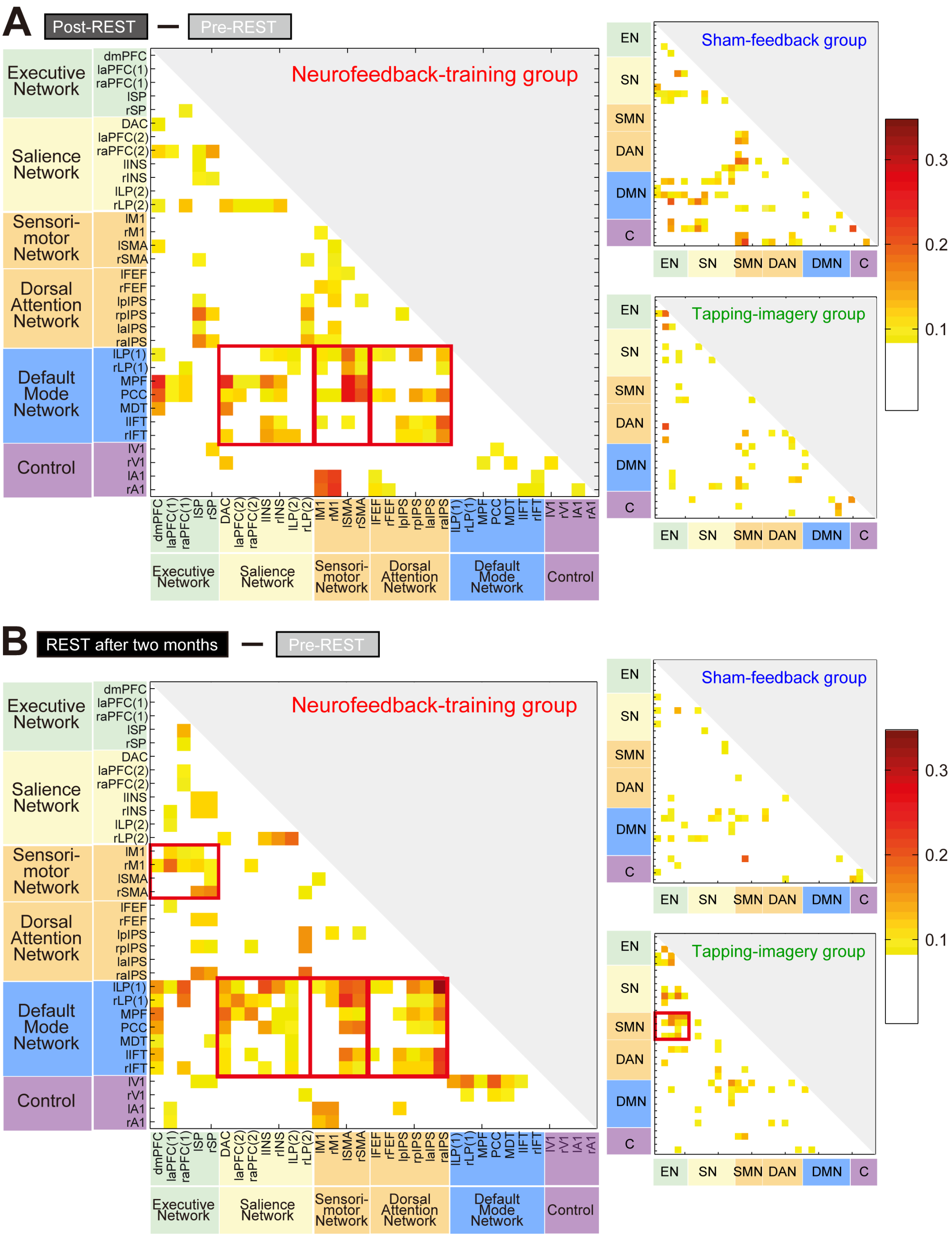

Supplementary Figure S5. Increase in correlation values from pre- to post-training in an

analysis using 33 ROIs. Seventeen ROIs were taken from the previous study investigating rs-fcMRI over the entire brain (Raichle, 2011) and added to the 16 ROIs in Table 1. Names of brain networks follow those in that previous study (Raichle, 2011). Each additional ROI is a spherical region of 7.5-mm radius centered on the MNI coordinates as listed in Table S2. Statistical analyses and graphic conventions follow those in Figure 5. Colored cells in the matrices indicate ROI pairs whose correlation values markedly increased (i.e. the increase in correlation value was greater than the summation of the averaged increase and standard deviation [mean + SD: -0.03 + 0.09 for Fig. S5A and -0.01 + 0.07 for Fig. S5B] across all pairs and subject groups). The red rectangles indicate network pairs having a significantly larger number of colored cells than that generated by a random process according to a bootstrap sampling approach ($P < 0.0001$; $P < 0.05$ after the Bonferroni correction; see Materials and Methods). Most of these network pairs were found in the neurofeedback training group with an increase from pre-REST to post-REST (**A**) as well as an increase from pre-REST to 'REST after two months' (**B**). Moreover, a consistent increase in correlation between network pairs from pre- to post-training (post-REST and REST after two months) was identified for the correlation between the DMN and dorsal-attention network (DAN), that between the DMN and salience network (SN), and that between the DMN and sensorimotor network (SMN). DAN includes lFEF, rFEF, lIPS and rIPS, each of which was included in the motor/visuospatial network (MVN) in our previous network analysis using 16 ROIs. The SMN includes lM1, rM1, lSMA and rSMA, each of which was included in the MVN. Therefore, although results were not exactly consistent between the 16-ROI and 33-ROI analyses, similar results (a consistent increase in correlation between regions related to the DMN or MVN) were observed in both analyses. Note that a significant increase was identified between DMN and SN, DMN and SMN, and DMN and DAN in the neurofeedback-training group regarding an increase from pre-REST to post-REST ($P = 0.0001$; $P < 0.05$ after the Bonferroni correction). A significant increase was identified between SMN and executive network (EN), DMN and SN, DMN and SMN, and DMN and DAN in the neurofeedback-training group as well as an increase between SMN and EN in the tapping imagery group regarding an increase from pre-REST to REST after two months ($P = 0.0001$; $P < 0.05$ after the Bonferroni correction).

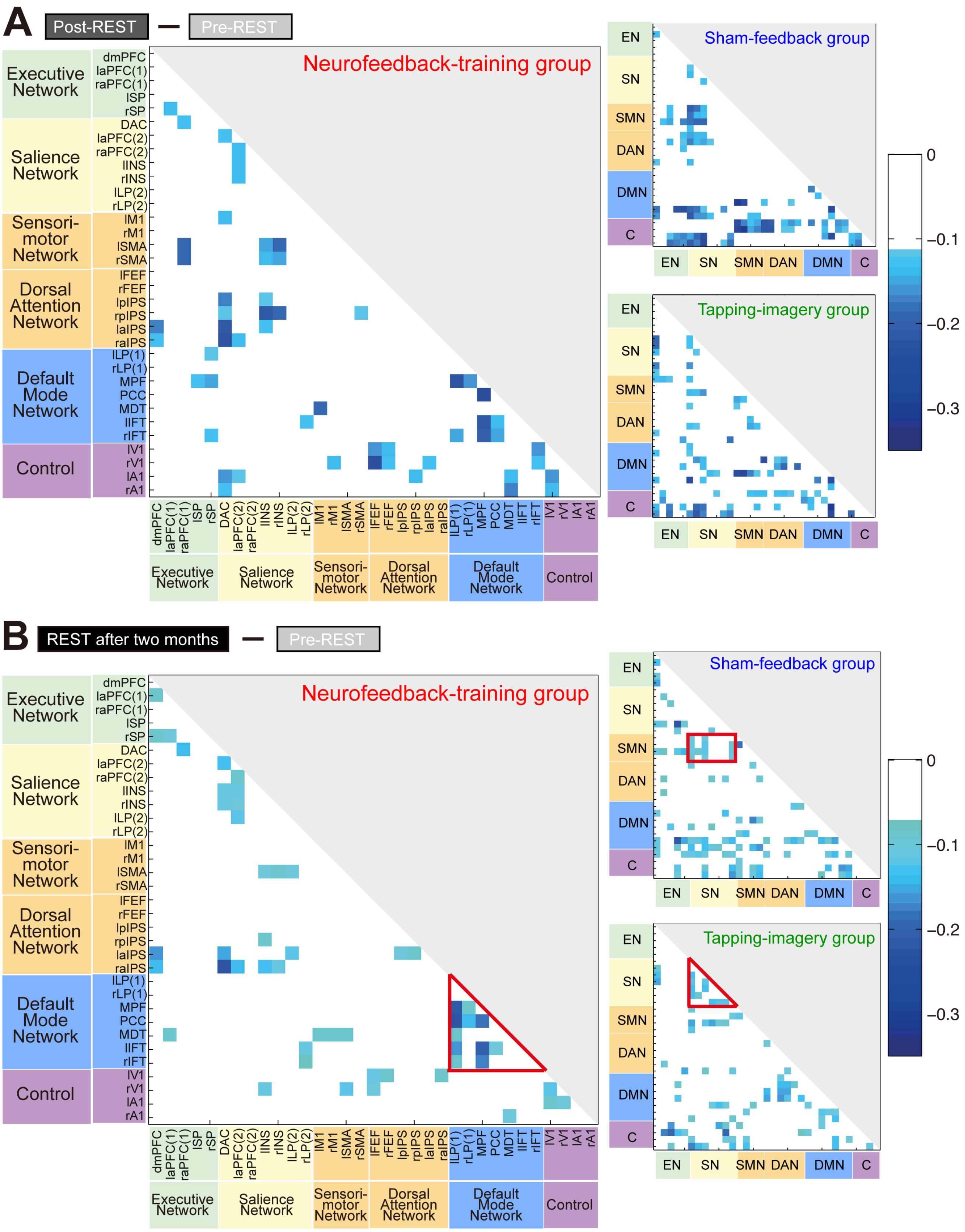

Supplementary Figure S6. Decrease in correlation values from pre- to post-training in an analysis using 33 ROIs. ROIs, statistical analyses and graphic conventions follow those in Figure S5

except for colored cells indicating ROI pairs whose correlations markedly decreased (more than mean - SD: -0.02 - 0.08 for Fig. S6A and -0.00 - 0.08 for Fig. S6B). The red triangle in Figure S6B indicates ROI combinations within DMN or salience network (SN) having a significantly larger number of colored cells than that generated by a random process according to a bootstrap sampling approach (decrease within DMN in neurofeedback group, $P = 0.0001$, decrease within SN in tapping imagery group, $P = 0.0001$; $P < 0.05$ after Bonferroni correction). The red rectangle indicates ROI combinations between sensorimotor network and SN in the sham-feedback group ($P = 0.0002$; $P < 0.05$ after correction). However, no combination of network pairs showed a significant decrease from pre-REST to either post-REST or REST after two months, which is consistent with the result of Figure S4.

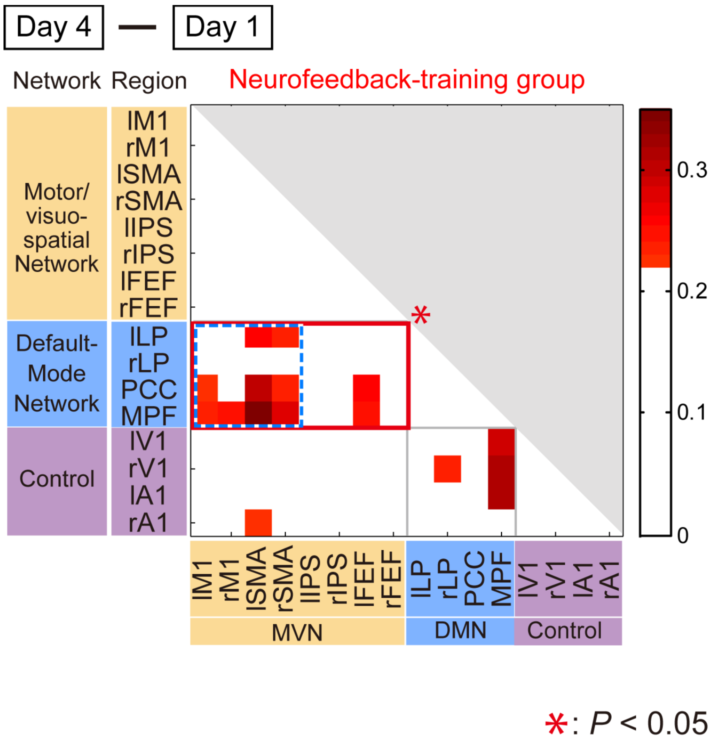

Supplementary Figure S7. Increase in z-transformed correlation values from day 1 to day 4 during neurofeedback training. We conducted post hoc analysis of fMRI data during neurofeedback training to investigate how correlated activity changed in regions other than the lM1- and lLP-ROIs in the neurofeedback training group. Motion correction and temporal filtering were the same as those used for real-time preprocessing (see "Online calculation of feedback score" in Materials and Methods). To investigate the increments of functional connectivity between days 1 and 4, we extracted the BOLD signal from 16 ROIs (see Table 1) during the imagery periods. The temporal correlation during the imagery period was computed for each region pair. By averaging the correlation coefficients across periods and trials within a day, we produced a correlation matrix containing all ROI pairs. We subtracted the z-transformed correlation matrix on day 1 from that on day 4 separately for each subject and then averaged the results across subjects in the neurofeedback training group. The averaged matrix was thresholded at the summation of mean and standard deviation (mean + SD: 0.13 + 0.09) across the cells. Colored cells in Figure S7 indicate cells whose values were larger than the threshold. Color bar indicates subtracted correlation value that is more than mean + SD. To identify combinations of network pairs (MVN-MVN, MVN-DMN, DMN-DMN,

MVN-control, DMN-control, and control-control) that possessed a significantly greater number of colored cells than that generated by a random process, we applied the bootstrap sampling method used for analysis of rs-fcMRI (see "Bootstrapping method for analysis of network correlation" in Materials and Methods) to the averaged matrix. We created bootstrap samples with neurofeedback training group data and computed threshold. Bootstrap sampling method indicated that the MVN and DMN (red rectangle) was the only network pair where a significant increment of functional connectivity was observed ($P = 0.002$; $P < 0.05$ after Bonferroni correction). Blue rectangle (dashed line) indicates region pairs between motor-related area and the DMN. r: right, l: left. See Table 1 for abbreviations of region names. These results suggest the following generalization processes from the increase in lM1-lLP correlation to that in MVN-DMN correlation: Tight connections within functional networks (Keller et al., 2011) induce regional co-modulation to cause generalization of the increased functional connectivity from M1-LP to (motor regions)-DMN, and then to MVN-DMN to some extent during neurofeedback training; then co-modulation was fully generalized to the entire MVN-DMN in the rest. Because motor regions are closely involved in similar brain functions and DMN regions are tightly connected with each other and other networks (Hagmann et al., 2008) the effect of neurofeedback training on the correlation between M1 and LP was probably first generalized across motor-related regions and the DMN during training (blue rectangle). Furthermore, in rs-fcMRI, this increase in correlation was generalized across many other region pairs between the MVN and DMN (Fig. 4B and red rectangles in Figs. 5A and 5B).

3. References

Fox, M. D. and Raichle, M. E. (2007). Spontaneous fluctuations in brain activity observed with functional magnetic resonance imaging. *Nat Rev Neurosci* 8, 700-11. doi: 10.1038/nrn2201.

Grezes, J. and Decety, J. (2001). Functional anatomy of execution, mental simulation, observation, and verb generation of actions: A meta-analysis. *Hum. Brain Mapp.* 12, 1-19.

Hagmann, P., Cammoun, L., Gigandet, X., Meuli, R., Honey, C. J., Wedeen, V. J., and Sporns, O. (2008). Mapping the structural core of human cerebral cortex. *PLoS Biol* 6, e159. doi: 10.1371/journal.pbio.0060159.

Keller, C. J., Bickel, S., Entz, L., Ulbert, I., Milham, M. P., Kelly, C., and Mehta, A. D. (2011). Intrinsic functional architecture predicts electrically evoked responses in the human brain. *Proc. Natl. Acad. Sci. U. S. A.* 108, 10308.

Laird, A. R., Fox, P. M., Eickhoff, S. B., Turner, J. A., Ray, K. L., McKay, D. R., Glahn, D. C., Beckmann, C. F., Smith, S. M., and Fox, P. T. (2011). Behavioral interpretations of intrinsic connectivity networks. *J. Cogn. Neurosci.* 23, 4022-37. doi: 10.1162/jocn_a_00077.

Raichle, M. E. (2011). The restless brain. *Brain connectivity* 1, 3-12. doi: 10.1089/brain.2011.0019.
